# Superior Mesenteric Artery Syndrome Secondary to Anorexia Nervosa and Methamphetamine Use

**DOI:** 10.7759/cureus.6121

**Published:** 2019-11-11

**Authors:** Brett M Johnson, Gopikrishna Paladugu

**Affiliations:** 1 Department of Internal Medicine, University of North Dakota School of Medicine and Health Sciences, Bismarck, USA; 2 Department of Internal Medicine, University of North Dakota School of Medicine and Health Sciences, Fargo, USA

**Keywords:** anorexia nervosa, superior mesenteric artery syndrome, small bowel obstruction, wilkies syndrome

## Abstract

Superior mesenteric artery (SMA) syndrome, or Wilkie syndrome, is a rare cause of small bowel obstruction due to compression of the duodenum between the SMA and aorta. Patients most at risk of SMA syndrome include those with rapid weight loss due to a variety of conditions including chronic illness, malignancy, trauma, HIV, eating disorders, substance abuse, or bariatric surgery. Characteristic radiologic findings include an aortomesenteric angle less than 25 degrees and an aortomesenteric distance of less than 8 mm. Symptoms are typically postprandial and notably include abdominal fullness, voluminous emesis, and abdominal pain. Here we present a case of SMA syndrome in a 19-year-old cachectic female who initially presented with sudden-onset nausea, vomiting, and severe abdominal pain. Imaging revealed a severely distend stomach and proximal duodenum with a transition point in the third portion of the duodenum consistent with SMA syndrome. Her symptoms resolved with nasogastric decompression in addition to fluid and electrolyte management. She later endorsed restrictive eating patterns consistent with anorexia nervosa as well as methamphetamine use for weight loss. She underwent close outpatient follow-up for her anorexia nervosa and substance abuse.

## Introduction

Superior mesenteric artery (SMA) syndrome, or Wilkie syndrome, is a rare cause of small bowel obstruction characterized by compression of the third segment of the duodenum between the SMA and aorta. SMA syndrome typically occurs when the retroperitoneal fat pad that supports the aortomesenteric angle decreases due to extreme weight loss, leading to duodenal compression [1]. This compression leads to a variety of symptoms depending on the severity of obstruction, but notably includes postprandial abdominal fullness, nausea, vomiting, and abdominal pain. While the exact incidence of SMA syndrome is not known, estimates range from 0.0024% to 0.34% in the general population [1,2]. Here we present a case of SMA syndrome in a young female secondary to anorexia nervosa and methamphetamine use that resolved with nasogastric decompression and supportive measures.

## Case presentation

A 19-year-old female was in her usual state of health until she developed sudden-onset nausea, vomiting, and abdominal pain. She had been unable to tolerate any oral intake without emesis. She subsequently sought treatment at an outside facility emergency department. Her history was notable for bipolar disorder and a 61 lb (27.7 kg) weight loss in the past year with about 30 lb (13.6 kg) lost in the prior month. Physical examination was notable for tachycardia at 105 beats per minute, a body mass index (BMI) of 19.05 kg/m^2^, and generalized abdominal tenderness. Initial laboratory evaluation revealed an elevated white blood cell count (WBC) at 18,600 per µL (normal limit: 4,000-11,000 per µL) with 16,400 per µL neutrophils (normal limit: 1,800-8,000 per µL) and a urine drug screen positive for methamphetamines, marijuana, and MDMA. A urinalysis was negative for nitrites or leukocyte esterase. Despite meeting systemic inflammatory response syndrome/sepsis criteria at this time, she did not receive further work-up or antibiotic treatment. She was given intravenous (IV) fluids and ondansetron with alleviation of her symptoms and tolerance of oral intake. She was subsequently discharged home.

She returned to the emergency department the following day with a recurrence of postprandial nausea, vomiting, and severe abdominal pain. She was again unable to tolerate any oral intake without emesis. Physical examination demonstrated a BMI of 18.03 kg/m^2^, decreased bowel sounds, and diffuse abdominal tenderness. She was given IV fluids and ondansetron with fentanyl for pain control. Repeat laboratory work was significant for an elevated WBC of 26,500 per µL. A lactic acid was normal at 1.0 mmol/L (normal limit: 0.4-2.0 mmol/L). Blood cultures were obtained, and a one-time dose of ceftriaxone was subsequently administered. A computed tomography (CT) scan of the abdomen and pelvis with intravenous contrast was obtained to assess possible intussusception as she was unable to tolerate any oral contrast.

**Figure 1:**
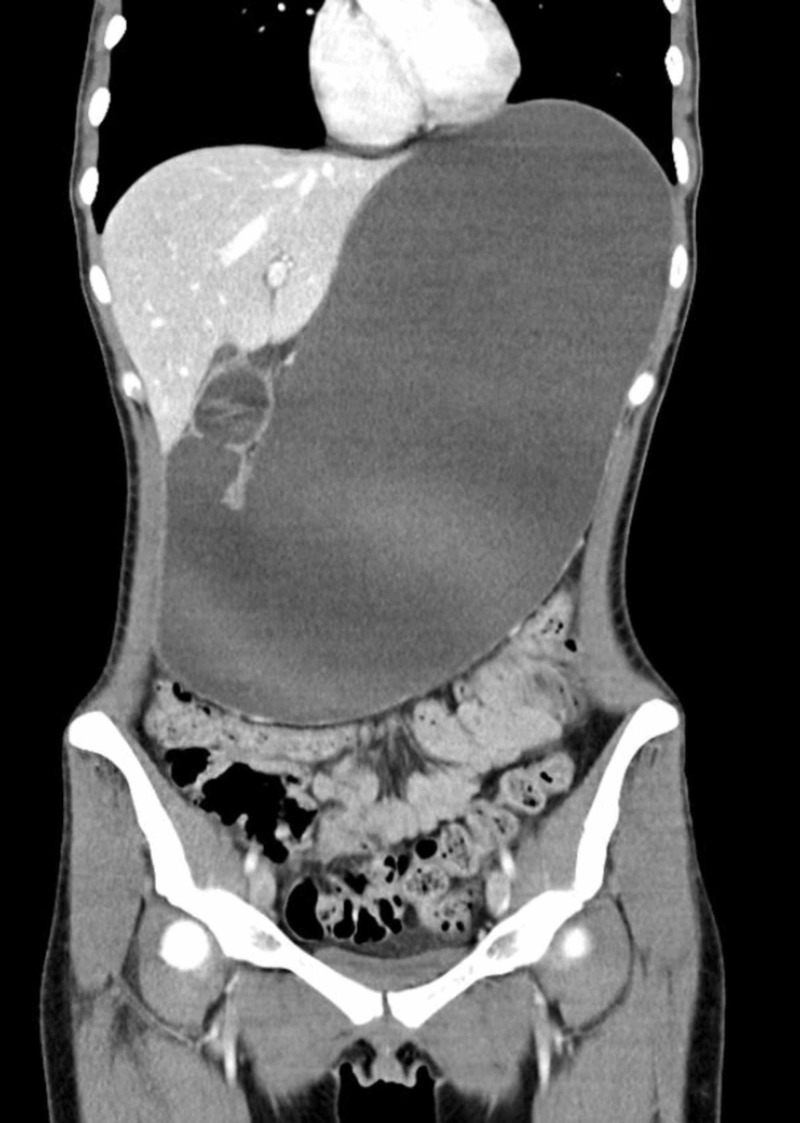
IV contrast-enhanced coronal computed tomography (CT) of the abdomen and pelvis consistent with small bowel obstruction. This figure demonstrates severe dilatation of the stomach and proximal duodenum consistent with small bowel obstruction. Portions of the distal small and large bowel are decompressed.

**Figure 2:**
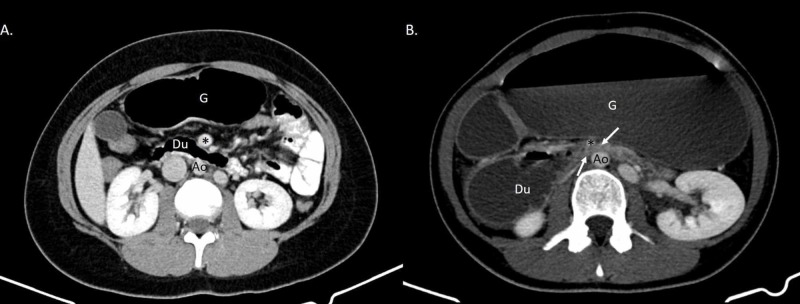
IV contrast-enhanced axial computed tomography (CT) of the abdomen and pelvis consistent with superior mesenteric artery syndrome, with eight-month comparison. Ao: aorta, asterisk: superior mesenteric artery (SMA), Du: duodenum, G: stomach. A. This figure demonstrates normal bowel eight months prior to the patient's acute presentation. It was performed due to right lower quadrant abdominal pain. B. This figure reveals dilated stomach and proximal duodenum with air fluid levels. There is an abrupt collapse of the third portion of the duodenum as it crosses posterior to the SMA close to midline. The arrows denote the transition point between dilated and collapsed small bowel.

**Figure 3:**
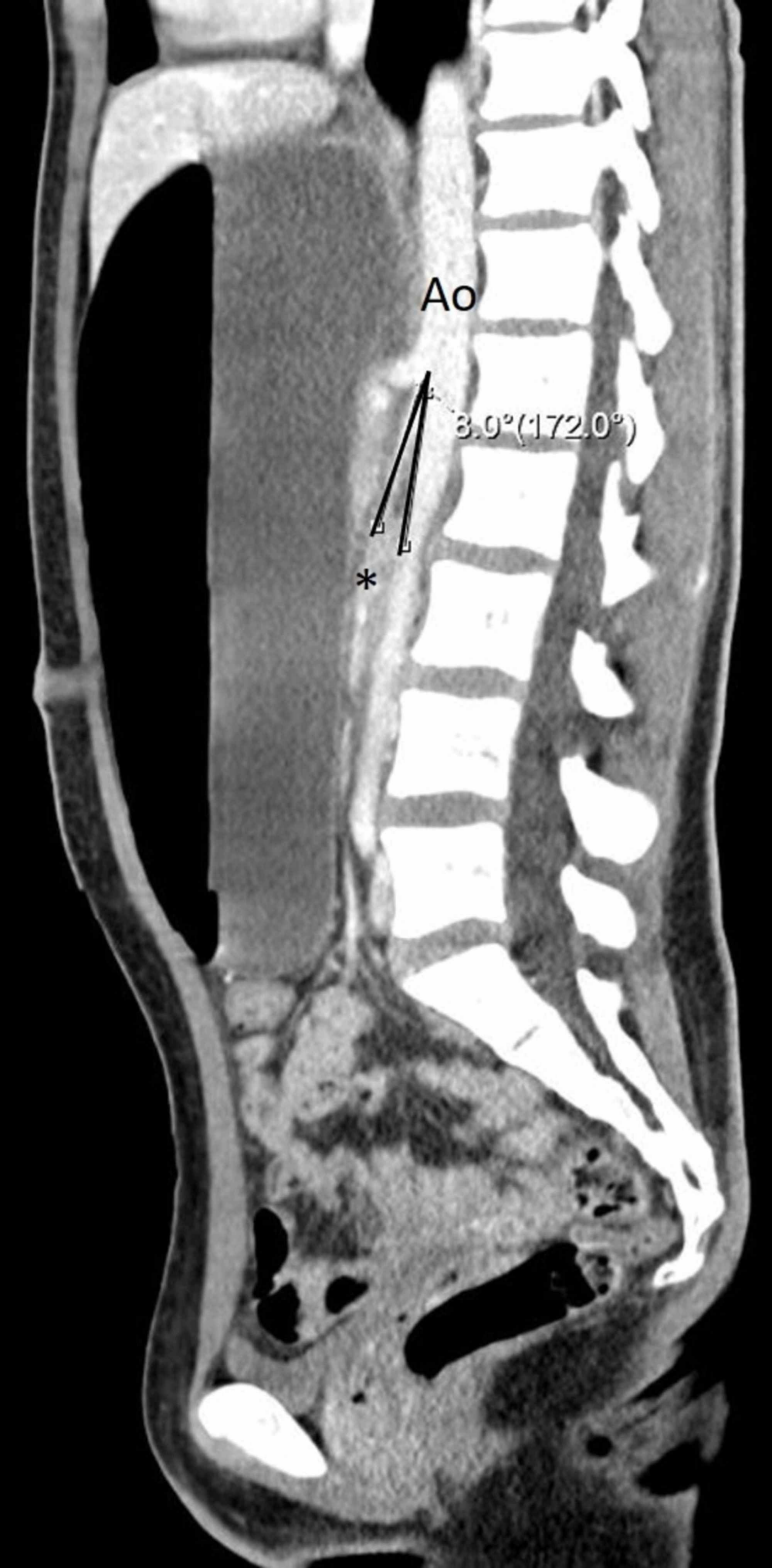
IV contrast-enhanced sagittal computed tomography (CT) of the abdomen and pelvis showing a reduced aortomesenteric angle of 8.0 degrees consistent with superior mesenteric artery syndrome. Ao: aorta, asterisk: superior mesenteric artery.

CT revealed a severely distended stomach and proximal duodenum with air-fluid levels and a transition point from dilated to decompressed duodenum at the midline as the third portion of the duodenum passed inferior to the SMA (Figures 1, 2). The aortomesenteric angle measured 8 degrees (normal limit: 38-65 degrees) with an aortomesenteric distance of 6.0 mm (normal limit: 10-28 mm) which was consistent with SMA syndrome (Figure 3).

She was transferred and admitted to the hospital for nasogastric decompression, fluid, and electrolyte support. The patient admitted to restrictive eating behavior in the months prior in addition to methamphetamine use for weight loss that psychiatry felt was consistent with anorexia nervosa. Gastroenterology performed an esophagogastroduodenoscopy, which revealed a dilated deformity of the first, second, and third portions of the duodenum. The fourth portion of the duodenum required abdominal pressure to advance the scope but was otherwise normal. Her symptoms began to improve throughout her hospital stay. On day 4 of her hospitalization, the nasogastric tube was discontinued, and her diet advanced as tolerated without return of her symptoms. She was discharged with close primary care and psychiatric follow-up. She returned to clinic one week after discharge and had already gained 5 lb (2.3 kg).

## Discussion

SMA syndrome was originally described by Rokitansky in 1842, who noted the SMA could compress the duodenum against the lumbar spine [3]. Wilkie later characterized the pathology in 1927 as postprandial epigastric fullness, early satiety, and voluminous emesis [4]. This led to the eponymous syndrome, although it is also known as arteriomesenteric duodenal compression, chronic duodenal ileus, or cast syndrome. Given its various names and ill-defined diagnosis, the exact incidence of SMA syndrome is difficult to determine. SMA syndrome is reported more frequently among older children and adolescents which could be related to rapid growth without corresponding weight gain [1,5,6].

Normally, the SMA originates from the abdominal aorta at approximately the level of the L1 vertebra. The SMA then runs anteroinferiorly, forming an acute angle in which the third segment of the duodenum rests. This aortomesenteric angle ranges from 38 to 65 degrees in humans standing erect and depends on retroperitoneal adipose and lymphatic tissue for support [1]. The aortomesenteric distance is typically 10 to 28 mm. The position of the duodenum in relation to the SMA is dictated by embryonic development. During the fifth week of gestation, the midgut herniates and rotates 270 degrees counterclockwise with the SMA as its primary axis. The midgut then reduces by the tenth week such that the SMA resides ventrally and caudally to the horizontal portion of the duodenum suspended by the ligament of Treitz [1,2].

Of the factors that can predispose an individual to SMA syndrome, major weight loss appears most frequently, especially if the BMI is below the third percentile [7-9]. Reported conditions that can lead to major weight loss, and thus SMA syndrome, include chronic cachectic conditions such as HIV, trauma, anorexia nervosa, and bariatric surgery. As the course of the SMA over the duodenum is a result of embryonic development, congenital factors and anatomic variation can also play a role in the development of SMA syndrome. These factors can include a shortened or hypertrophic ligament of Treitz, short mesenteric root, low origin of the SMA, adhesions, and Ladd’s bands [2]. SMA syndrome can also be acquired post-operatively, with scoliosis surgery reported most commonly [7-10]. In this patient, restrictive eating patterns consistent with anorexia nervosa and methamphetamine use was the cause of her rapid weight loss, and thus her case of SMA syndrome. Despite her reported 27 kg weight loss, her BMI was still at the fifth percentile.

SMA syndrome occurs when the third segment of the duodenum is compressed by the SMA against the aorta. The resulting compression leads to either partial or complete small intestinal obstruction with a plethora of symptoms that can occur in either an acute or chronic setting. One case series noted common symptoms that include vomiting (70%), nausea (66.3%), abdominal pain (65%), anorexia (33.8%), and postprandial fullness (33.8%) [11]. Our patient exhibited all of these commonly reported symptoms in the acute setting with the most prominent initial symptom of vomiting. Her symptoms were also worse postprandially, fitting the classical disease description. However, our patient experienced more generalized abdominal pain than some of the other reported cases which may be due to the severity of her bowel dilatation.

Given the clinically non-specific findings found with SMA syndrome, diagnosis rests on imaging modalities. The most prominent diagnostic finding of SMA syndrome is when the aortomesenteric angle is below 25 degrees. CT imaging typically results in the best view, although endoscopy or endoscopic ultrasound can also play a role [12-14]. CT has the added benefit of demonstrating the transition point of obstruction in relation to the superior mesenteric vessels and the aorta [11]. The Haynes’ criteria serve as a general guide for the radiologic diagnosis of SMA syndrome and include: 1) dilatation of the duodenum proximal to the obstruction, 2) abrupt vertical cutoff in the third portion of the duodenum, 3) anti-peristaltic flow of contrast proximal to the obstruction, 4) delay in gastroduodenojejunal transit time of four to six hours, and 5) relief of obstruction on bringing the knees to the chest [14]. While the Haynes' criteria incorporate the use of oral contrast, our patient was unable to tolerate it due to the severity of her symptoms. Despite the lack of oral contrast in our case, we were still able to diagnose SMA syndrome due to the characteristic reduction of the aortomesenteric angle and distance in conjunction with small bowel obstruction.

The initial treatment of SMA syndrome centers on decompression of the obstruction and replacement of any fluids or electrolytes. Increasing the intravascular space can temporarily increase the aortomesenteric angle and improve symptoms. Additionally, nutritional therapy is vital to replace the fat pad loss that predisposed the individual to SMA syndrome in the first place. Enteral feeding is preferred over total parenteral nutrition and can include routes such as nasojejunal tube or jejunostomy. Encouragement of oral intake as soon as tolerated following decompression can also accelerate the return to normal weight [2,12]. Our patient responded well to nasogastric decompression with fluid and electrolyte management. We were also able to advance her diet to enteral feedings within four days as her symptoms subsided. Importantly for our patient, close outpatient management was scheduled with both her primary care provider and psychiatry in order to manage her anorexia nervosa and substance abuse in order to prevent recurrence.

If the SMA syndrome remains refractory to decompression and nutrition after a few weeks, surgical options can be considered. These can include duodenojejunostomy or Treitz ligament division (Strong’s operation) [12-15]. Current practice favors laparoscopic duodenojejunostomy due to a success rate of 80% to 100% in addition to laparoscopic benefits of minimal blood loss, less postoperative pain, better cosmetic outcomes, earlier recovery of normal peristalsis, and reduced hospital stays [2,16].

## Conclusions

SMA syndrome can be a diagnostically challenging condition with life-threatening consequences if not quickly treated. A low threshold of suspicion is required in order to ensure swift management. Prompt decompression, in addition to fluid and nutritional support, can improve symptoms and prevent the disease from recurring. However, if symptoms persist, a surgical approach should be employed. After the patient is stabilized, long-term management of the etiology of SMA syndrome becomes paramount, especially with chronic conditions such as anorexia nervosa or substance abuse.

## References

[1] Mathenge N, Osiro S, Rodriguez II, Salib C, Tubbs RS, Loukas M (2014). Superior mesenteric artery syndrome and its associated gastrointestinal implications. Clin Anat.

[2] Mandarry MT, Zhao L, Zhang C, Wei ZQ (2010). A comprehensive review of superior mesenteric artery syndrome. Eur Surg.

[3] Rokitanski CV (1842). Lehrbuch der Pathologischen Anatomie.

[4] Wilkie DPD (1927). Chronic duodenal ileus. Am J Med Sci.

[5] Laffont I, Bensmail D, Rech C, Prigent G, Dizien O (2002). Late superior mesenteric artery syndrome in paraplegia: case report and review. Spinal Cord.

[6] Bin Waqar SH, Khan AA, Mohiuddin O (2019). Perplexing case of Wilkie's syndrome: a rare presentation in a young patient. Cureus.

[7] Berchi FJ, Benavent MI, Cano I, Portela E, Urruzuno P (2001). Laparoscopic treatment of superior mesenteric artery syndrome. Pediatr Endosurg Innov Tech.

[8] Shekar PA, Ramdev P, Chauhan V, Rao C (2019). Superior mesenteric artery syndrome: a rare complication following left radical nephrectomy and IVC thrombectomy. Urology.

[9] Sahni S, Shiralkar M, Mohamed S (2019). Superior mesenteric artery syndrome: the dark side of weight loss. Cureus.

[10] Tsirikos A, Anakwe RE, Baker ADL (2008). Late presentation of superior mesenteric artery syndrome following scoliosis surgery: a case report. J Med Case Reports.

[11] Lee TH, Lee JS, Jo Y (2012). Superior mesenteric artery syndrome: where do we stand today?. J Gastrointest Surg.

[12] Naseem Z, Premaratne G, Hendahewa R (2015). “Less is more”: non operative management of short term superior mesenteric artery syndrome. Ann Med Surg.

[13] Applegate GR, Cohen AJ (1988). Dynamic CT in superior mesenteric artery syndrome. J Comput Assist Tomogr.

[14] Unal B, Aktas A, Kemal G (2005). Superior mesenteric artery syndrome: CT and ultrasounagraphy findings. Diagn Interv Radiol.

[15] Suhani AL, Ali S, Jhaketiya A, Thomas S (2014). Short and hypertrophic ligament of Treitz: a rare cause of superior mesenteric artery syndrome. J Clin Diagn Res.

[16] Ganss A, Rampado S, Savarino E, Bardini R (2019). Superior mesenteric artery syndrome: a prospective study in a single institution. J Gastrointest Surg.

